# Effects of Phytoremediation Treatment on Bacterial Community Structure and Diversity in Different Petroleum-Contaminated Soils

**DOI:** 10.3390/ijerph15102168

**Published:** 2018-10-02

**Authors:** Yuanyuan Shen, Yu Ji, Chunrong Li, Pingping Luo, Wenke Wang, Yuan Zhang, Daniel Nover

**Affiliations:** 1 Key Laboratory of Subsurface Hydrology and Ecological Effects in Arid Region, Ministry of Education, School of Environmental Science and Engineering, Chang’an University, Xi’an 710064, China; shenyuan1982@xawl.edu.cn (Y.S.); 2016129049@chd.edu.cn (Y.J.); changanl@chd.edu.cn (C.L.); Wenkew@chd.edu.cn (W.W.); 18766953412@163.com (Y.Z.); 2 School of Biological and Environmental, Xi’an University, Xi’an 710065, China; 3 Engineering Research Center for Groundwater and Eco-Environment of Shaanxi Province, Xi′an 710054, Shaanxi, China; 4 School of Engineering, University of California—Merced, Merced, CA 95343, USA; dnover@ucmerced.edu

**Keywords:** petroleum-contaminated soil, bacterial diversity, community structure, phytoremediation, high-throughput sequencing

## Abstract

Increased exploitation and use of petroleum resources is leading to increased risk of petroleum contamination of soil and groundwater. Although phytoremediation is a widely-used and cost-effective method for rehabilitating soils polluted by petroleum, bacterial community structure and diversity in soils undergoing phytoremediation is poorly understood. We investigate bacterial community response to phytoremediation in two distinct petroleum-contaminated soils (add prepared petroleum-contaminated soils) from northwest China, Weihe Terrace soil and silty loam from loess tableland. High-throughput sequencing technology was used to compare the bacterial communities in 24 different samples, yielding 18,670 operational taxonomic units (OTUs). The dominant bacterial groups, *Proteobacteria* (31.92%), *Actinobacteria* (16.67%), *Acidobacteria* (13.29%) and *Bacteroidetes* (6.58%), increased with increasing petroleum concentration from 3000 mg/kg–10,000 mg/kg, while *Crenarchaeota* (13.58%) and *Chloroflexi* (4.7%) decreased. At the order level, *RB41*, *Actinomycetales, Cytophagales, envOPS12, Rhodospirillales, MND1* and *Xanthomonadales,* except *Nitrososphaerales*, were dominant in Weihe Terrace soil. Bacterial community structure and diversity in the two soils were significantly different at similar petroleum concentrations. In addition, the dominant genera were affected by available nitrogen, which is strongly associated with the plants used for remediation. Overall, the bacterial community structure and diversity were markedly different in the two soils, depending on the species of plants used and the petroleum concentration.

## 1. Introduction

Soil quality is associated with agricultural production and the quality of human life. Petroleum-related industrial activities lead to extensive soil pollution and, as a result its toxicity to organisms and human health, petroleum has been heavily studied. Petroleum products, including diesel, gasoline, and lubricants, can be released to the environment through spills, accidents, as unintended by-products of industrial activities, leading to diffuse and local pollution [1,2]. Petroleum extraction, refining and consumption all represent significant pathways for contamination in soil and sediment by total petroleum hydrocarbons (TPHs) [3,4]. When soil is heavily polluted, groundwater contamination, as well as changes in soil composition and structure can occur [5,6]. Environmental contamination is widespread due to the great number of facilities and processes, with consequences for human health, ecosystems and other receptors [7,8]. Soil composition and structure also exert strong effects on soil microbial communities, which play a significant role in the fate of petroleum-sourced contaminants. It is necessary to characterize microbial communities in contaminated soils to better understand treatment options and to develop improved strategies for reducing harmful substances present in the environment and suggest approaches for manipulating soil microorganisms to enhance these effects.

Although many studies of petroleum-contaminated soils have been conducted, few clearly demonstrate interactions between the microbial community structure at the genus level and the environment. Petroleum-contaminated soil can modify the bacterial community structure [9,10,11], and petroleum can act as a substrate for certain bacteria, driving transformation and soil remediation. To improve petroleum degradation efficiency, plants such as *Acorus calamus*, *Betula pendula*, cultivars of willow (*Salix*) and *Cynodon dactylon* have been used in phytoremediation studies [12,13,14,15]. The rhizosphere comprises the surface of the roots and the surrounding soil area where plant root exudates sustain a high microbial activity and high microbial density. Plants mediate a rhizosphere effect illustrated by plant-specific microbial communities. Generally, the rhizosphere of plants harbors a higher diversity of bacteria than the surrounding bulk soil due to root exudates and oxygen that stimulate bacteria [16]. The combined application of soil microorganisms and plant species can significantly decrease PAH (polycyclic aromatic hydrocarbon) concentrations, increase microbial populations and enhance the efficiency of bioremediation [17,18,19,20,21]. However, community structure and diversity in many environments remain unstudied. Various studies have focused on microbial populations and microbial communities at the phylum level, reporting the Shannon index and using rarefaction curve analysis and phylogenetic trees [22,23,24]. Sequencing approaches have influenced the characterization of bacterial community structure and diversity in soils at the phylum level. Non-sequencing-based molecular methods, such as denatured gradient gel electrophoresis (DGGE), may produce inadequate conclusions in comparison to direct sequencing analysis techniques [25,26]. Comparisons of DGGE and high-throughput barcode sequencing have been performed in many studies [27,28,29], and the results show that high-throughput barcode sequencing has substantial advantages in accuracy and cost, offering an excellent tool for studying microbial communities [30,31].

This study used high-throughput sequencing on the Illumina MiSeq platform to explore bacterial community structure and diversity using different phytoremediation techniques in two distinct soils with four different concentrations of petroleum. Specifically, phytoremediation model plants *Cynodon dactylon* (L.) Pers. and *Agropyron cristatum* (L.) Gaertn were applied to contrast undisturbed plant communities in Weihe Terrace soil (Soil 0) and silty loam from Loess tableland (Soil 1). We compared the response of bacterial communities to two different soils with different reclamation plants and contamination levels. This study elucidates the effects of petroleum, soil environment and plants on bacteria. In addition, the results provide insight into microbial community structure and diversity in polluted soils [32]. 

## 2. Materials and Methods

### 2.1. Site Description

Experiments were performed at the Water and Environment In-situ Experimental Area on Weishui Campus, Chang’an University, Xi’an, China. The urban area of Xi’an is located at 34°16′ N 108°56′ E and lies on the Guanzhong Plain surrounded by eight rivers and streams in the south-central part of Shaanxi province. The average elevation of Xi’an city is 400 m, and the mean annual precipitation is about 553 mm. The Wei River Valley is characterized by hot summers, cold and dry winters and dry springs and autumns.

### 2.2 Experimental Design and Sample Collection

**Figure 1:**
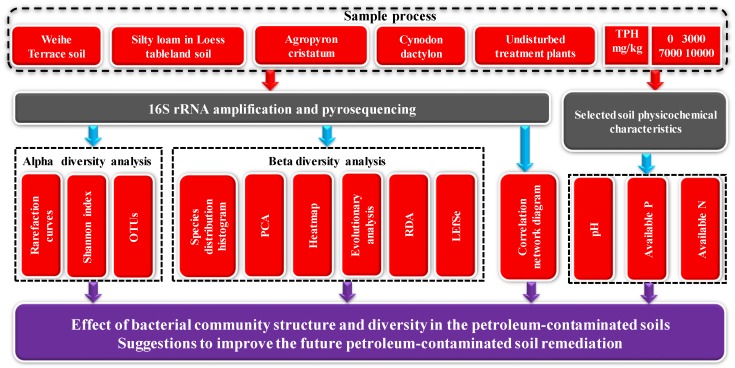
Framework of the analysis processes (TPH: total petroleum hydrocarbons; OTUs: operational taxonomic units; PCA: principal component analysis; RDA: redundancy analysis).

This study’s research framework is presented in Figure 1. We selected Weihe Terrace soil and silty loam from the loess tableland as study soils and, we investigate the promotion and toxicity of petroleum to plants and microbes [33,34]. We experimentally investgate remediation of soils with different petroleum concentrations (i.e., 0, 3000, 7000 and 10,000 mg/kg, respectively) using the phytoremediation grass plants *Agropyron cristatum* (L.) Gaertn. and *Cynodon dactylon* (L.) Pers. The different soils, plants and total petroleum hydrocarbon (TPH) of samples were compared with respect to their bacteria community using Alpha and Beta diversity analysis and correlation network diagrams. The results inform applicability of different remediation techniques to different polluted environments and yield suggestions for future soil remediation efforts.

Test seeds were purchased from the Eastern Grassland Livestock Technology Company in Beijing, China. Soil (0–20 cm) was collected from the Weishui Campus, Chang’an University (Weihe Terrace) and Sickle-Bay Township in Yan-an City (silty loam from Loess tableland). The two soils reflect different particle compositions and physical and chemical properties (Table S1). The composition of sand, silt and clay particles in Weihe Terrace soils were 21.8%, 69.6% and 9.6%, respectively. Silty loam from loess tableland was 90.8% silt and 9.2% clay. Petroleum was added to the soils and test soils were prepared using different petroleum concentrations: 0 (control), 3000, 7000 and 10,000 mg/kg. The TPHs of soils were based on U.S. Environmental Protection Agency Standard 3550 [35]. The test site had 24 test plots (Table 1) with plot dimensions of 1 m (L) × 1 m (W) × 0.9 m (H). Samples (0–20 cm soils) were collected from 24 test plots of two different soils with different reclamation plants and contamination levels. Each sample was divided into two parts: one for microbial analysis and one that was sieved (<2 mm) to analyze physical and chemical properties. Soil pH was measured using a pH meter with a 1:5 (wt/vol) ratio of soil to water following shaking for 30 min. Soil physicochemical analyses of these habitats are described in detail in previous research [36].

### 2.3. DNA Extraction, 16S rRNA Amplification and Pyrosequencing

DNA extraction was conducted using an Ezup genomic DNA extraction kit for soil (Sangon Biotech, Xi’an, China, Cat# SK8264). DNA concentration and quality were checked using a NanoDrop Spectrophotometer (Beijing Pu Analytical General instrument Co., Ltd., Beijing, China). Extracted DNA was diluted to 10 ng/μL and stored at −40 °C for subsequent use.

Universal primers 515F (5′-GTGCCAGCMGCCGCGGTAA-3′) and 806R (5′-GGACTACHVGGGTWTCTAAT-3′) with 12-nt unique barcodes were used to amplify the V4 hypervariable region of the 16S rRNA gene for pyrosequencing using a MiSeq sequencer [37,38]. The PCR mixture (25 μL) contained 1× PCR buffer and 1.5 mM MgCl_2_, with each deoxynucleoside triphosphate at 0.4 μM, each primer at 1.0 μM along with 0.5 U of Ex Taq (TaKaRa, Dalian, China) and 10 ng soil genomic DNA. The PCR amplification program included initial denaturation at 94 °C for 3 min, followed by 30 cycles of 94 °C for 40 s, 56 °C for 60 s and 72 °C for 60 s and a final extension at 72 °C for 10 min. Two PCR reactions for each sample were run and combined after PCR amplification. PCR products were subsequently subjected to electrophoresis using 1.0% agarose gel. Correct-sized bands were excised and purified using a SanPrep DNA Gel Extraction Kit (Sangon Biotech, China, Cat# SK8132) and quantified with NanoDrop. All samples were pooled together with equal molar amounts from each sample. The sequencing samples were prepared using TruSeq DNA kits (Illumina, CA, USA) according to manufacturer instructions. The purified library was diluted, denatured, re-diluted, mixed with PhiX (equal to 30% of the final DNA amount) as described in the Illumina library preparation protocols and applied to an Illumina MiSeq system for sequencing with the Reagent Kit v2 2 × 250 bp, as described in the manufacturer’s manual.

### 2.4 Analysis of Pyrosequencing Date

The sequence data were processed using QIIME Pipeline-Version 1.7.0 (US National Ecological Observatory Network) (http://qiime.org/). All sequence reads were trimmed and assigned to each sample based on their barcodes. High quality sequences (length >150 bp, without ambiguous base ‘N’ and average base quality score >30) were used for downstream analysis. Sequences were clustered into operational taxonomic units (OTUs) at a 97% identity threshold. The aligned ITS (internal transcribed spacer) gene sequences were used for chimera checks using the UCHIME (algorithm for detecting chimeric sequences, implemented in the uchime ref and uchime denovo commands) algorithm [39]. All samples were randomly resampled to 18,670 reads. We calculated alpha-diversity, including phylogenetic distance, Chao1 (Chao1 is an index used to estimate the number of OTUs in a community and total number of species), estimator of richness, observed species, Shannon’s diversity index, and performed beta-diversity analyses (i.e., RDA, Heatmap), for which the rarefaction curves were generated from the observed species. Taxonomy was assigned using the Ribosomal Database Project classifier [40].

### 2.5. Nucleotide Sequence Accession Numbers

The sequences of the 24 soil samples in this study were deposited in the GenBank/NCBI databases under Accession Numbers RUN8782479–RUN8782502.

## 3. Results

### 3.1. This Physicochemical Characteristics of the Soil

It was necessary to analyze physical and chemical soil properties to describe the relationship between the parameters and the microbial community accurately (Table 2). We compared samples from all 24 plots comprised of combinations of two distinct soils, contamination levels and reclamation plants. In general, soil organic matter (SOM) content showed significant differences, especially between the different types of soil. Overall, the contents of SOM in the Weihe Terrace soil were higher than the contents in silty loam from Loess tableland. Soil pH ranged from 8.35–8.97 across all sites. The Weihe Terrace soil pH was slightly lower, more suitable for plant cultivation. Highest available potassium was highest in non-polluted phytoremediation soil regardless of soil type. Table 2 shows that plant type had a clear effect on available nitrogen, available phosphorus and available potassium. The soils with *A. cristatum* and *C. dactylon* plants had lower available phosphorus and available potassium compared to undisturbed control plot soil. Available nitrogen in soil with *C. dactylon* was at least three-times higher than available nitrogen in soil with *A. cristatum*, which was roughly two-times lower than the available nitrogen in the control soil. The level of available nitrogen in soil, therefore, depends strongly on the plant community.

### 3.2. Bacterial Community Structure and Diversity

Table 3 shows the rarefaction curve displaying diversity across the 24 samples, with coverage of the 24 sample sequences ranging from 0.81–0.86. In addition, 5110–6059 OTUs at a 97% similarity level represented cultured environmental clones. The rarefaction curves and Shannon–Wiener curves were generally flat, with a decreasing slope (Figure S1). Even when the sequencing number increased, few new OTUs were generated, indicating that high-throughput sequencing coverage can reflect bacterial diversity in soil. Community richness was evaluated using the Chao1 index. Comparing Weihe Terrace soil and silty loam from the Loess tableland, microbial richness was affected more by the change of petroleum concentration and reclamation plants than different soil types (Table 3). Moreover, the Shannon diversity indices indicated that the samples from 1B3, 1B7, and 1B10 had lower diversity than the other samples.

**Figure 2:**
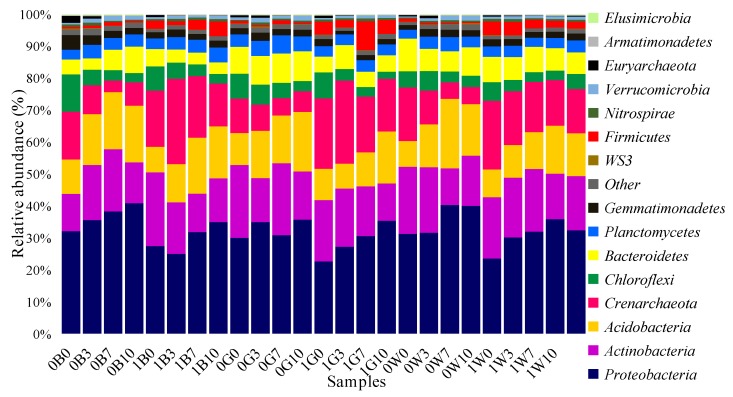
Relative read abundance of dominant bacterial community structure at the phyla level at each site. Relative abundances (>1%) are based on the proportional frequencies of those DNA sequences that could be classified at the phyla level.

Within all samples, there were 52 phyla, 173 classes, 341 orders, 559 families and 912 genera. Figure 2 shows the community structure in all 24 samples categorized by the most common phyla (i.e., *Proteobacteria, Actinobacteria, Acidobacteria, Crenarchaeota, Chloroflexi, Bacteroidetes, Planctomycetes, Gemmatimonadetes, WS3, Firmicutes, Nitrospirae, Verrucomicrobia, Euryarchaeota, Armatimonadetes, Elusimicrobia* and unclassified phyla). At the phylum level, the microbial community structure was related to changes in soil petroleum concentration and soil type (Figure 2). *Proteobacteria* made up 23–34% of all communities in the silty loam from the Loess tableland, but was present at significantly higher percentages in all communities of the Weihe Terrace soil (31–40%). In contrast, the abundances of both *Actinobacteria* and *Acidobacteria* in the two soils were similar. In previous studies, *Proteobacteria* and *Acidobacteria* were reported to be the dominant groups in petroleum-contaminated soils from oilfields and paddy fields [41,42,43]. For all groups, soil type and petroleum concentrations influenced bacterial community abundance, with *Proteobacteria* and *Acidobacteria* dominant at higher soil TPH levels in our study (total petroleum hydrocarbons: 7000 mg/kg, 10,000 mg/kg). The dominated bacterial population increased when the petroleum concentration increased from 0 mg/kg–10,000 mg/kg, e.g. see *Proteobacteria* and *Acidobacteria* in Figure 2. Although the mechanism for this increase is unclear, at 10,000 mg/kg, the populations of *Chloroflexi* and *Crenarchaeota* decreased.

Among the 341 orders, *Nitrososphaerales, iii1-15, RB41, Actinomycetales, Cytophagales, envOPS12, Rhodospirillales, MND1* and *Xanthomonadales* were found as shown in Figure S2. *Nitrososphaerales* and *RB41* were the dominant orders in the 24 samples. *Nitrososphaerales* made up 2.7–16.4% of the total culture communities in the Weihe Terrace soil, less than levels in the silty loam from the Loess tableland (i.e., 12.9–26.1%). Thus, silty loam from Loess tableland appears to be more suitable for *Nitrososphaerales* compared to Weihe Terrace soil. In contrast, relative abundances of *RB41, Actinomycetales, Cytophagales, envOPS12* and *Rhodospirillales* were favorably represented in the Weihe terrace soil. Uncultured members of the order *Cytophagales* have been reported in water and in high tidal flats [44,45]. *Cytophagales* was rarely reported as the dominant bacterial order in petroleum-contaminated soil under phytoremediation treatment.

**Figure 3:**
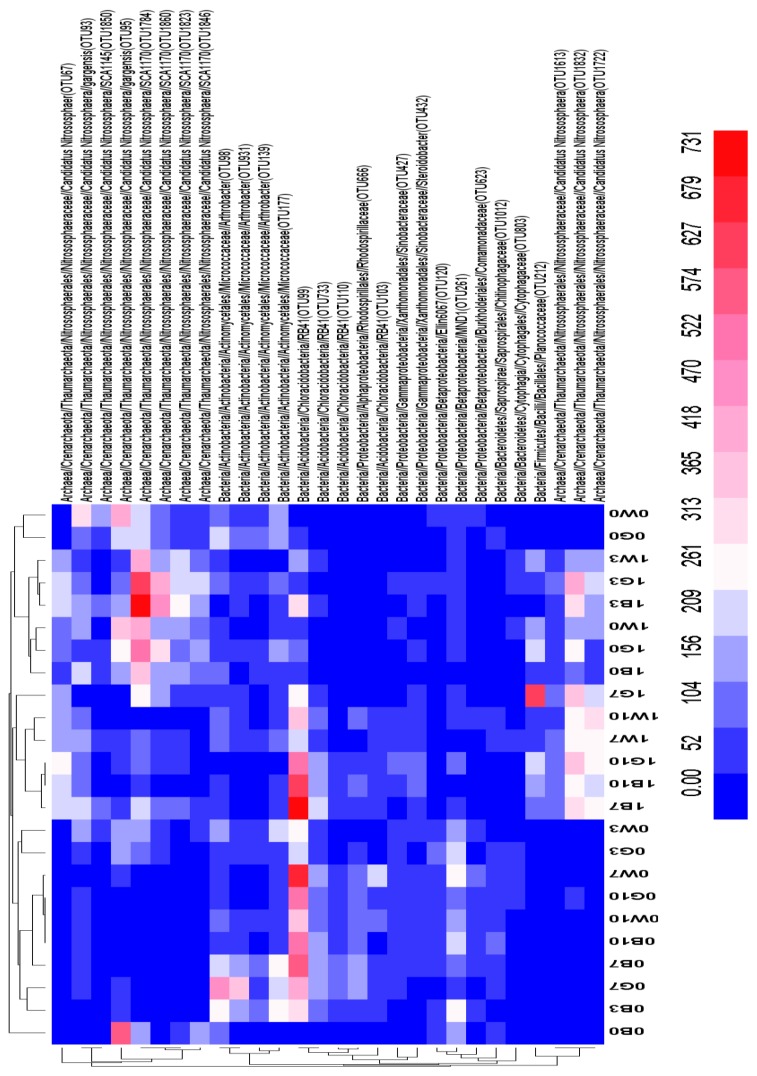
Heatmap of 24 samples based on the correlation between environmental factors and the abundance of dominant groups. The color intensity of the scale represents the microbial community relative abundance with respect to the abundance of each OTU relative to all bacterial sequences.

The scale bar in Figure 3 represents the percent abundances within each OTU. The colors in the heat map show the dominant groups in the 24 samples. At the phylum level, *Proteobacteria* was the prevalent group, while *Betaproteobacteria, Gammaproteobacteria and Alphaproteobacteria. Crenarchaeota, Actinobacteria* and *Acidobacteria* were also common groups. *RB41* was discovered in 18 samples, but few were found in 0B0, 0W0, 1B0, 1W0, 0G0 and 1G0. *SCA1170* was also common, indicated by deep red in the heat map. We found that *Planococcaceae, Candidatus* and *Nitrososphaera* were always present in the silty loam of the Loess tableland, but *MND1* was rare in this soil type.

**Figure 4:**
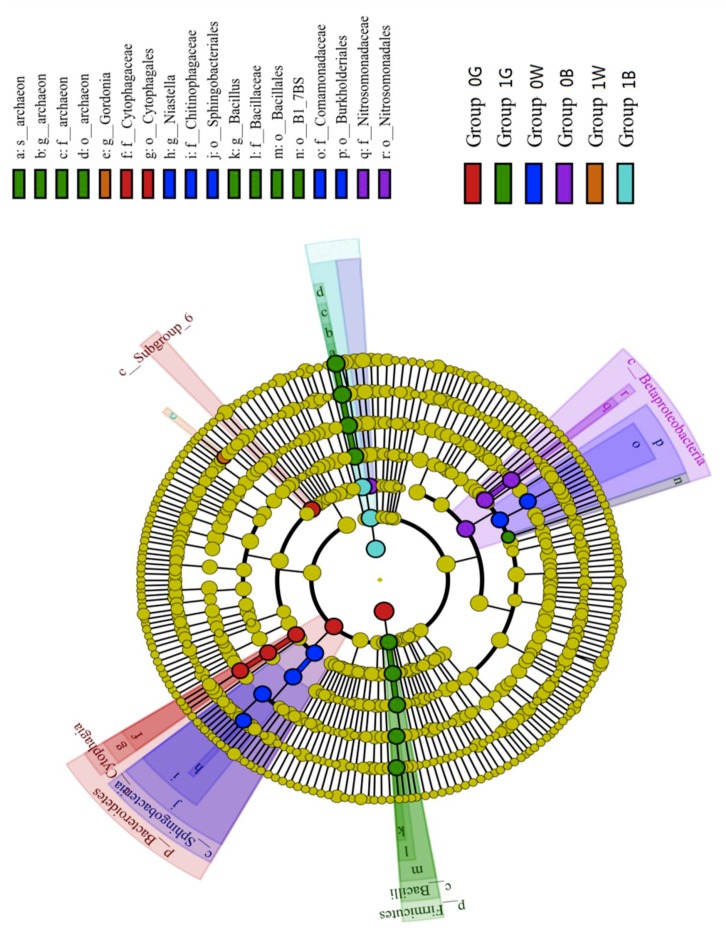
LEfSe analysis of evolutionary branching graphs. The circle of evolutionary branching maps from internal to external radiation represents the classification level from the phyla to the species; the principle of coloring is that the species with no significant differences are colored yellow, and the other species are colored according to the group with the highest abundance of the species; Groups 0 and 1: the reclamation in Weihe Terrace soil and silty loam in Loess tableland, respectively; Groups B, G and W: the reclamation plants of *Agropyron cristatum* (L.) Gaertn, *Cynodon dactylon* Linn. Pers. and undisturbed plants.

In addition to the heatmap (Figure 3), we performed linear discriminant analysis (LDA) and effect size analysis (Figure 4). The circle of evolutionary branching maps from internal to external represents the classification level from phyla to species. The principle of coloring is that the species with no significant differences are colored yellow, and the other species are colored according to the group with the highest abundance. The length of the histogram represents the size of the difference in species. The groups were divided by *C. dactylon*, *A. cristatum* and undisturbed plants in two distinct soils (i.e., Group 0B, Group 0G, Group 0W, Group 1B, Group 1G, Group 1W) with different colors. LEfSe analysis (linear discriminant analysis) of evolutionary branching graphs showed that in the microbial communities of two distinct soils and with different reclamation plants, there was a marked difference in bacterial community and diversity between Groups 0G and 1G, 0B and 1B, 0W and 1W. Groups 0G and 0W are in the *Bacteroidetes* phylum, *Cytophagaceae* family and the genus *Niastella*. Similarly, Groups 1G and 1B belong to *Firmicutes* at the phylum level. Groups 0B and 1W are part of *Nitrospirae* and *Actinobacteria*, and Group 1W is of the genus *Gordonia*. The two distinct soils and reclamation plants exerted influence over bacterial community structure and diversity.

### 3.3. Relationships of Soil Environmental Factors with Soil Bacterial Diversity and Community Composition

**Figure 5:**
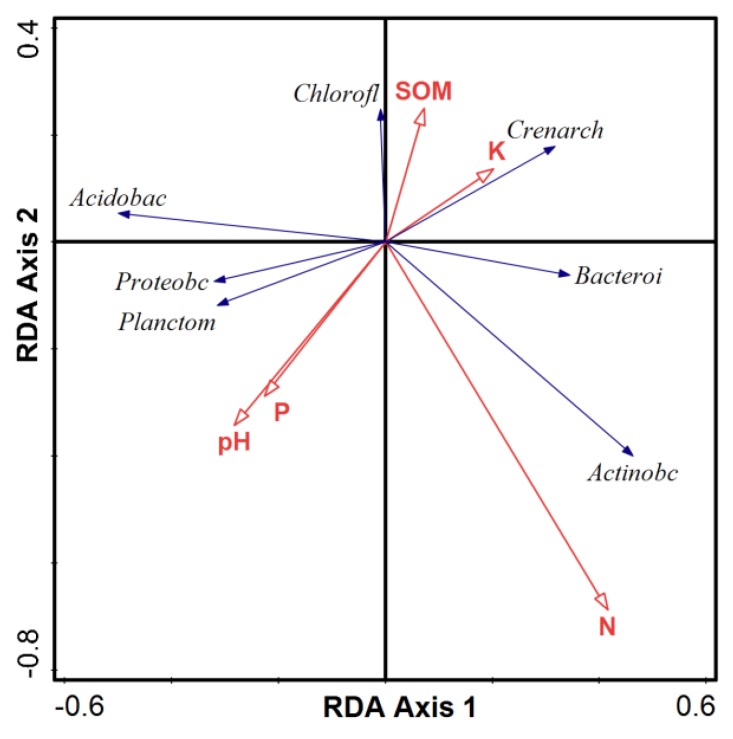
Redundancy analysis (RDA) for seven primary soil bacterial communities (*Proteobacteria, Actinobacteria, Acidobacteria, Crenarchaeota, Chloroflexi, Bacteroidetes, Planctomycetes*) from all samples associated with environmental variables (pH, SOM, available P, available N and available K).

The relationship between bacterial community and physicochemical soil properties was analyzed using detrended correspondence analysis PCA (Figure S3). The longest gradient length was less than three, indicating that samples are best assessed using RDA (Figure 5). The cumulative percentage variance of species and PCA analysis was 38.86% and 21.79%, respectively. RDA was used to assess bacterial community and soil properties, including factors such as pH, SOM, available phosphorus, available potassium and available nitrogen. According to the angle between arrows, the approximated correlation was determined to be positive when an acute angle was observed, while an angle greater than 90 degrees indicated a negative value. Available nitrogen had a strong positive relationship with Actinobacteria, but a negative relationship with Acidobacteria and Chloroflexi. This result corresponds with the dominant bacterial community structure at the phyla level (Figure 2) and previous studies [46,47]. Available nitrogen affected the abundance of Acidobacteria and Chloroflexi. In addition, pH and available phosphorus had a slight impact on Crenarchaeota and Chloroflexi. Similarly, the presence of Crenarchaeota and Chloroflexi had positive relationships with SOM and available potassium, but negative relationships with Proteobacteria, Acidobacteria and Planctomycetes.

## 4. Discussion

### 4.1. Differences in Bacteria Diversity among 24 Samples

In our study, *Proteobacteria, Acidobacteria* and *Bacteroidetes* represented a substantial component of the bacterial community structure, consistent with the findings of Fernando et al. [48], who noted that *Acidobacteria* and *Bacteroidetes* were capable of degrading petroleum in contaminated soils. In all reclamation sites, *Proteobacteria* and *Actinobacteria* were dominant, which was especially true for Weihe Terrace soil compared to silty loam from the Loess tableland. Silty loam from the Loess tableland is relatively less favorable for the growth of plants and microorganisms in the phytoremediation of contaminated soil because it typically has a smaller particle size, with larger surface area and smaller particle diffusion distance compared to Weihe Terrace soil. This limits oxygen transfer and decomposition by microorganisms [49]. In general, the bacterial community is related to both the physical and chemical properties of soil and the soil quality. At the order level, the relative abundance of all classified sequences higher than 0.01 is shown in Figure S2. The dominant *Nitrososphaerales* was well represented in silty loam from Loess tableland, compared to the Weihe Terrace soil, indicating that *Nitrososphaerales* was the more suitable bacteria in silty loam from Loess tableland. Regarding remediation plants, only *Nitrososphaerales* in the soil planted with *A. cristatum* showed a clear distinction between petroleum-contaminated soil and undisturbed soil.

High contamination levels saw large increases in proportions of *Proteobacteria* and *Acidobacteria*. Our results are also consistent with the finding that high TPH concentrations in soil are often associated with the presence of certain microorganisms [50,51,52]. Because petroleum is a carbon source for bacteria in soils, a certain concentration of petroleum provides enough carbon for microorganisms and makes bacteria more active. This also explains why less dominant bacteria (*Proteobacteria* and *Acidobacteria)* are seen in the no petroleum samples compared to petroleum-contaminated soils. Several studies have reported that *Chloroflexi* thrive in alkaline arid soils like our pilot sites [53,54]. However, we found that *Chloroflexi* was present in relatively lower concentrations in high hydrocarbon contamination areas, exerting an adverse effect on populations. Similarly, the higher concentrations of petroleum (7000 mg/kg and 10,000 mg/kg) were less favorable than lower concentrations at the order level for *MND1* and *Xanthomonadales*, which is consistent with previous research on *Xanthomonadales* [55]. *Xanthomonadales* are highly effective degraders at lower contamination levels. At a lower concentration of 0 mg/kg and 3000 mg/kg, petroleum can nourish Chloroflexi, *MND1* and *Xanthomonadales,* while high contamination levels are intolerable for these bacteria. This may be because high concentrations of petroleum adhere to the surface of the plant’s roots, hindering root respiration and reducing the production of rhizosphere exudates, which make them difficult to grow dominant bacteria. Although few studies have reported the presence of *Crenarchaeota* in petroleum-contaminated soil, this phylum accounted for a quarter of the total bacterial population among all plots tested. *Cytophagales* also represent unreported taxa with respect to community structure in petroleum-contaminated soils. This shows that *Crenarchaeota* and *Cytophagales* are very important in the study of petroleum pollution, a finding that demands further study.

### 4.2. Response of Bacteria to Different Physicochemical Properties Soil

Phytoremediation can cause changes to soil fertility, bacterial community structure and diversity [56,57]. Soil type and species of reclamation plants have important effects on soil fertility and bacterial species richness. Fertility index analysis showed that soil organic matter was different in the two soil types. Soil fertility was higher in Weihe Terrace soil that had higher SOM content than the silty loam from the Loess tableland. In a previous study, Zhang [58] suggested that petroleum soil contaminants had no significant effect on available nitrogen, available phosphorus and available potassium. However, during phytoremediation attempts in petroleum-contaminated soil, we observed that soil nutrient levels were strongly associated with reclamation plants. Compared with undisturbed soils, available phosphorus and potassium were lower in phytoremediation plots. Remediation plants completely used the soil nutrients and plants altered the profile of rhizosphere microorganisms, promoting bacterial decomposition, suggesting the response of soil microbial communities and TPH dissipation in the rhizosphere [59,60]. Additionally, soil fertility, microbial species and plants had a strong influence on available nitrogen, which showed large variation among samples. Available nitrogen for plots of *C. dactylon* (G) and undisturbed plots (W) were triple and double that of *A. cristatum* (B), respectively. Zeng et al. [61] previously showed that nitrogen fertilization directly affects soil bacterial diversity and indirectly affects bacterial community composition, but direct or indirect interactions among plants, soils and microbes in response to nitrogen were not clear. As the imbalanced C:N:P ratio is a limiting factor in oil degradation [62], phytoremediation could stimulate the nitrogen cycle-related metabolic activity and degrading capacity of functional groups.

Several studies have demonstrated that bacterial species richness and abundance are associated with physical and chemical properties of soil [63,64]. As nitrogen is usually the most important growth factor for plants and soil microorganisms [65], we analyzed bacterial species richness and diversity indices combined with physical and chemical properties of soil to perform RDA (Figure 5). The results demonstrated that available nitrogen was closely linked with bacterial community structure and diversity. Available nitrogen had a positive impact on *Actinobacteria, Crenarchaeota* and *Bacteroidetes*, as well as other dominant bacteria, while pH, soil organic matter, available phosphorus and available potassium factors were also important for regulating bacterial distribution. For example, pH and available phosphorus contributed to *Proteobacteria* and *Planctomycetes*, while SOM might be related to *Chloroflexi* and available potassium to *Crenarchaeota* prevalence. This indicates that pH and available phosphorus had similar influences on soil microbial communities. The small change in pH from 8.35–8.97 drove a significant difference in community structure, as seen in Figure 5. Overall, the bacterial community appeared to be sensitive to slight variations in environment factors. Thus, the results provide insights into the microbial community composition in these different ecosystems and identified the main factors shaping this composition, which will lead to a more comprehensive understanding of microbial distribution in ecosystems.

### 4.3. The Bacteria Relationship in the Soils

The relationship between different bacteria in 24 samples was clarified [66] based on high-throughput sequencing techniques. We used the SparCC algorithm to conduct group correlation analysis in all sites combined, and the 50 bacterial genera with the highest correlation are shown in Figure S4. Each circle represents a species, and the size of the circles represents the species abundance. The strength of correlation between two species is indicated using line weight and color. Wide lines are associated with the strength of the correlation. When the line color is orange, a positive correlation is indicated. Green line color stands for a negative correlation. Bacterial populations (i.e., *Bacterium, archaeon, Nitrososphaerales, RB41, Terrimonas*) affect each other, as shown in Figure S4. *Archaeon* had positive relationships with *Nitrososphaerales,* but negative relationships with *Bacterium,* and *RB41* had an antagonistic effect on *archaeon.* This correlation analysis from the 24 samples can be used as a reference for the dynamics of microbial species in other petroleum-contaminated soils.

## 5. Conclusions

Major components of bacterial communities of soil plots with varied TPH contamination and phytoremediation approaches were identified by high-throughput sequencing technology. Compared with Weihe Terrace soil, we found that silty loam soil from Loess tableland did not favor phytoremediation. The higher petroleum concentration soils favored bacterial richness except for *Crenarchaeota* and *Chloroflexi*, the abundance of which was related to available nitrogen, SOM and available potassium. Remediation plants changed the general physical and chemical properties of soil, especially available nitrogen. Thus, petroleum concentration, remediation plants, as well as soil type had important effects on bacterial community structure and diversity. The results suggest that the addition of different nitrogen sources following phytoremediation in petroleum-contaminated soils may improve remediation outcomes by promoting a favorable microbial community structure. It is also necessary to analyze soil microbial community structure regularly during phytoremediation. The diversity of petroleum-degrading bacteria functional genes (i.e., LmPH, alkb, CAT, P450, C23O, amoA, narG) is also worthy of further study.

## Figures and Tables

**Table 1:** Total petroleum hydrocarbon (TPH) treatments for Weihe Terrace soil (Soil 0) and silty loam in Loess tableland (Soil 1).

Samples		0 (0 mg/kg TPH)	3 (3000 mg/kg TPH)	7 (7000 mg/kg TPH)	10 (10,000 mg/kg TPH)
Soil 0	B	0B0	0B3	0B7	0B10
	G	0G0	0G3	0G7	0G10
	W	0W0	0W3	0W7	0W10
Soil 1	B	1B0	1B3	1B7	1B10
	G	1G0	1G3	1B7	1G10
	W	1W0	1W3	1W7	1W10

*Notes:***B**: soil with *Agropyron cristatum*; **G**: soil with *Cynodon dactylon*; **W**: undisturbed plants’ soil.

**Table 2:** Physical and chemical properties of soils for each site.

Number	pH	SOM (g/kg)	A. P (g/kg)	A. N (g/kg)	A. K (g/kg)	Number	pH	SOM (g/kg)	A. P (g/kg)	A. N (g/kg)	A. K (g/kg)
0B0	8.47 ± 0.03	0.66 ± 0.02	22.06 ± 0.13	3.36 ± 0.05	109.58 ± 0.18	1B0	8.52 ± 0.09	0.44 ± 0.02	14.70 ± 0.3	3.34 ± 0.03	84.54 ± 1.2
0G0	8.97 ± 0.04	0.80 ± 0.01	24.32 ± 0.09	13.33 ± 0.02	118.37 ± 0.2	1G0	8.58 ± 0.2	0.53 ± 0.04	16.42 ± 0.02	14.34 ± 0.09	49.94 ± 1.1
0W0	8.35 ± 0.03	0.48 ± 0.01	62.77 ± 1.21	7.20 ± 0.11	166.17 ± 2.6	1W0	8.58 ± 0.01	0.48 ± 0.02	16.60 ± 0.06	4.21 ± 0.06	120.06 ± 0.98
0B3	8.46 ± 0.02	0.72 ± 0.04	21.11 ± 0.8	2.48 ± 0.02	100.47 ± 1.6	1B3	8.61 ± 0.06	0.44 ± 0.02	14.92 ± 0.1	3.81 ± 0.03	79.54 ± 0.76
0G3	8.56 ± 0.02	0.79 ± 0.02	21.21 ± 0.4	12.66 ± 0.04	97.77 ± 0.9	1G3	8.62 ± 0.03	0.59 ± 0.01	12.29 ± 0.09	12.24 ± 0.11	72.78 ± 1.32
0W3	8.44 ± 0.06	0.69 ± 0.05	25.36 ± 0.07	5.58 ± 0.09	160.11 ± 4.1	1W3	8.51 ± 0.05	0.33 ± 0.03	20.48 ± 0.12	6.73 ± 0.04	93.00 ± 3.1
0B7	8.38 ± 0.05	0.66 ± 0.03	16.07 ± 0.04	3.52 ± 0.01	128.92 ± 1.0	1B7	8.64 ± 0.07	0.59 ± 0.05	17.76 ± 0.02	4.76 ± 0.06	97.24 ± 0.27
0G7	8.42 ± 0. 02	0.66 ± 0.01	24.65 ± 1.01	17.10 ± 0.07	116.56 ± 3.2	1G7	8.60 ± 0.01	0.61 ± 0.02	9.00 ± 0.13	12.24 ± 0.06	91.82 ± 0.11
0W7	8.45 ± 0.11	0.59 ± 0.02	39.47 ± 1.33	6.40 ± 0.03	162.66 ± 1.7	1W7	8.59 ± 0.05	0.30 ± 0.04	20.73 ± 0.25	5.88 ± 0.05	101.26 ± 0.25
0B10	8.46 ± 0.06	0.76 ± 0.04	25.86 ± 1.2	5.24 ± 0.07	114.16 ± 3.4	1B10	8.63 ± 0.08	0.65 ± 0.01	11.34 ± 0.06	5.81 ± 0.02	104.10 ± 0.83
0G10	8.48 ± 0.2	0.98 ± 0.01	12.40 ± 0.08	12.87 ± 0.02	103.60 ± 2.3	1G10	8.57 ± 0.2	0.65 ± 0.05	8.24 ± 0.02	15.79 ± 0.06	83.38 ± 0.16
0W10	8.42 ± 0.02	0.79 ± 0.03	38.22 ± 0.83	6.71 ± 0.06	131.82 ± 1.62	1W10	8.59 ± 0.01	0.42 ± 0.02	10.04 ± 0.15	6.56 ± 0.21	85.42 ± 0.18

Notes: SOM, soil organic matter; A. N, available nitrogen; A. P, available phosphorus; A. K, available potassium. Values designate different oil pollution concentrations and plants in Weihe Terrace soil (Soil 0) and silty loam in Loess tableland (Soil 1); B, G and W: the reclamation plants of *Agropyron cristatum*, *Cynodon dactylon* and undisturbed plants, respectively; original TPH 0, 3, 7, and 10: 0 mg/kg (control), 3000 mg/kg, 7000 mg/kg, and 10,000 mg/kg, respectively.

**Table 3:** Diversity indices calculated based on a cutoff of 97% similarity of 16S rRNA sequences of 18,670 reads per sample.

ID	OTU97%	Coverage	Richness and Diversity Indices		
			Chao1	Shannon	Simpson
0B0	5110	0.86	8298.92	10.94	0.9976
0B3	5612	0.83	10,476.23	10.98	0.9977
0B7	5913	0.82	11,961.23	11.16	0.9978
0B10	5579	0.83	10,726.74	10.95	0.9973
1B0	5886	0.81	11,811.36	11.07	0.9979
1B3	6055	0.81	12,231.10	10.81	0.9955
1B7	5742	0.82	11,235.88	10.75	0.9961
1B10	5671	0.82	11,281.68	10.89	0.9970
0G0	5989	0.82	11,029.75	11.32	0.9986
0G3	5879	0.82	11,645.19	11.33	0.9988
0G7	5437	0.84	10,218.77	10.93	0.9973
0G10	6158	0.81	11,373.13	11.41	0.9983
1G0	5743	0.82	11,134.00	10.92	0.9971
1G3	5649	0.82	10,752.91	10.73	0.9960
1G7	5738	0.82	11,503.65	10.82	0.9965
1G10	5581	0.83	11,237.97	10.86	0.9971
0W0	5460	0.84	10,085.13	10.98	0.9979
0W3	6059	0.81	11,672.13	11.29	0.9985
0W7	5682	0.83	11,198.93	11.06	0.9974
0W10	5764	0.82	11,294.68	11.21	0.9985
1W0	5886	0.82	11,429.94	11.10	0.9976
1W3	5804	0.83	10,674.72	11.12	0.9980
1W7	5755	0.82	11,214.03	11.05	0.9981
1W10	5696	0.83	10,848.33	11.06	0.9979

Notes: OTU97%: operational taxonomic units; Chao1: the estimated bacterial richness values; Coverage: Good’s nonparametric coverage estimator; Shannon: nonparametric Shannon diversity index; Simpson: nonparametric Simpson diversity index.

## Supplementary Materials

The following are available online at http://www.mdpi.com/1660-4601/15/10/2168/s1: Table S1: General physical and chemical properties for Weihe Terrace soil (Soil 0) and silty loam in Loess tableland (Soil 1), Figure S1: Rarefaction analyses of samples (a) and Shannon—Wiener curves of samples (Rarefaction curves of OTUs clustered at 97% sequence identity across different soil samples. Soil 0 and Soil 1 respectively represent the Weihe Terrace silty and loam soil and silty loam in Loess tableland. B, G and W: the reclamation plants of *Agropyron cristatum* (L.) Gaertn, *Cynodon dactylon* Linn. Pers and undisturbed plants, respectively; original TPH 0, 3, 7 and 10: 0 mg/kg (control), 3000 mg/kg, 7000 mg/kg, and 10,000 mg/kg, respectively.), Figure S2: Community structure in all samples at the order level (relative abundances (>0.2%) are based on the proportional frequencies of those DNA sequences that could be classified at the order level), Figure S3: Multiple-sample PCA analysis of 24 samples associated with bacterial community and physicochemical soil. Figure S4: Correlation network diagram at the genera level (Circles represent specific species, and the size of circles stands for the abundance. The correlation between two species is described as line weight and color. The wide lines explain the strength of correlation. The orange lines are a positive correlation, and green stands for negative correlation. Screening data with a greater correlation group (>0.1) draw a common expression analysis network diagram at the genera level.).
