# Supplementary material for: Effects of Phytoremediation Treatment on Bacterial Community Structure and Diversity in Different Petroleum-Contaminated Soils
Source: Int J Environ Res Public Health. 2018 Oct 2;15(10):2168. doi: 10.3390/ijerph15102168 (PMC6211031; doi:10.3390/ijerph15102168)
Supplement: Supplementary file 1 [file ijerph-15-02168-s001.pdf]

Supplementary Material

Effects of Phytoremediation Treatment on Bacterial Community Structure and Diversity in Different Petroleum-contaminated Soils

Yuanyuan Shen [1,2,3] Yu Ji [1], Chunrong Li [1,*] , Pingping Luo [1*], Wenke Wang[1], Yuan Zhang [1], Daniel Noverd[4]

[1] Key Laboratory of Subsurface Hydrology and Ecological Effects in Arid Region，Ministry of Education, School of Environmental Science and Engineering, Chang'an University，Xi'an, 710064 China;

[2] School of Biological and Environmental, Xi'an University，Xi'an, Shaanxi, 710065 China;

[3] Engineering Research Center for Groundwater and Eco-Environment of Shaanxi Province，Xi'an Shaanxi, 710054

[4] School of Engineering, University of California – Merced, Merced, CA, 95343, USA

* Correspondence: Chunrong Li E-mail: changanl@chd.edu.cn, Telephone: +862982339951; Pingping Luo Email: lpp@chd.edu.cn, Telephone: +862982339376

Received: date; Accepted: date; Published: date

Table S1. General physical and chemical properties for Weihe terrace soil (Soil 0) and silty loam in loess tableland (Soil 1)

Name	Soil particle composition/%			Soil chemical composition analysis Ω (B)/10−2							Oxidation Reduction Potential/ mv	Conductivity /μs·cm−1
	Sand	Silt	clay	SiO_2	Al_2O_3	Fe_2O_3	CaO	MgO	K_2O	Na_2O		
Weihe terrace soil (Soil 0)	21.8	69.6	9.6	67.4	10.91	3.05	5.26	1.41	2.45	2.08	170	175
silty loam in loess tableland (Soil 1)	-	90.8	9.2	62.6	11.21	3.95	6.03	1.61	2.15	1.82	174	181

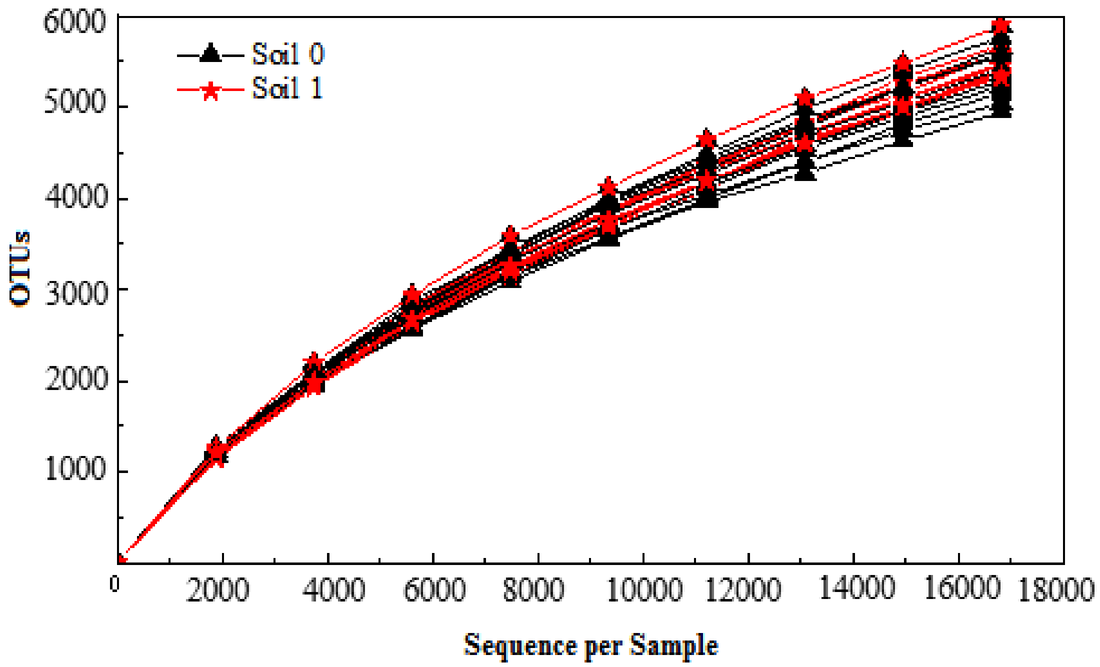

(a)

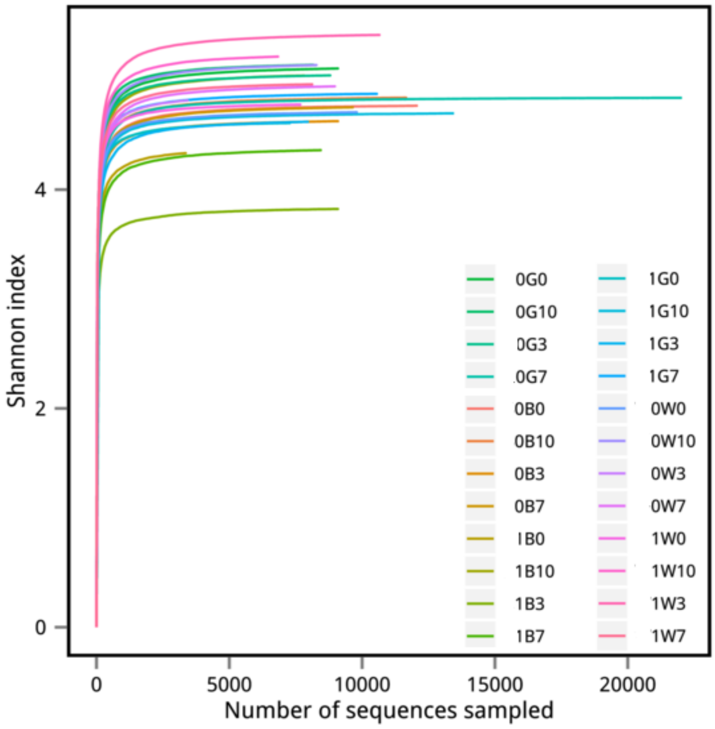

(b)

Fig. S1. Rarefaction analyses of samples (a) and Shannon Wiener curves of samples.(rarefaction curves of OTUs clustered at 97% sequence identity across different soil samples. Soil 0 and soil1 respectively represent the Wei he Terrace silty and loam soil silty loam in Loess Tableland. B, G and W: the reclamation plants of Agropyron cristatum (L.) Gaertn, Cynodondactylon Linn.Pers, and undisturbed plants, respectively; original TPH 0, 3, 7, and 10: 0 mg/kg (control), 3,000 mg/kg, 7,000 mg/kg, and 10,000 mg/kg, respectively.)

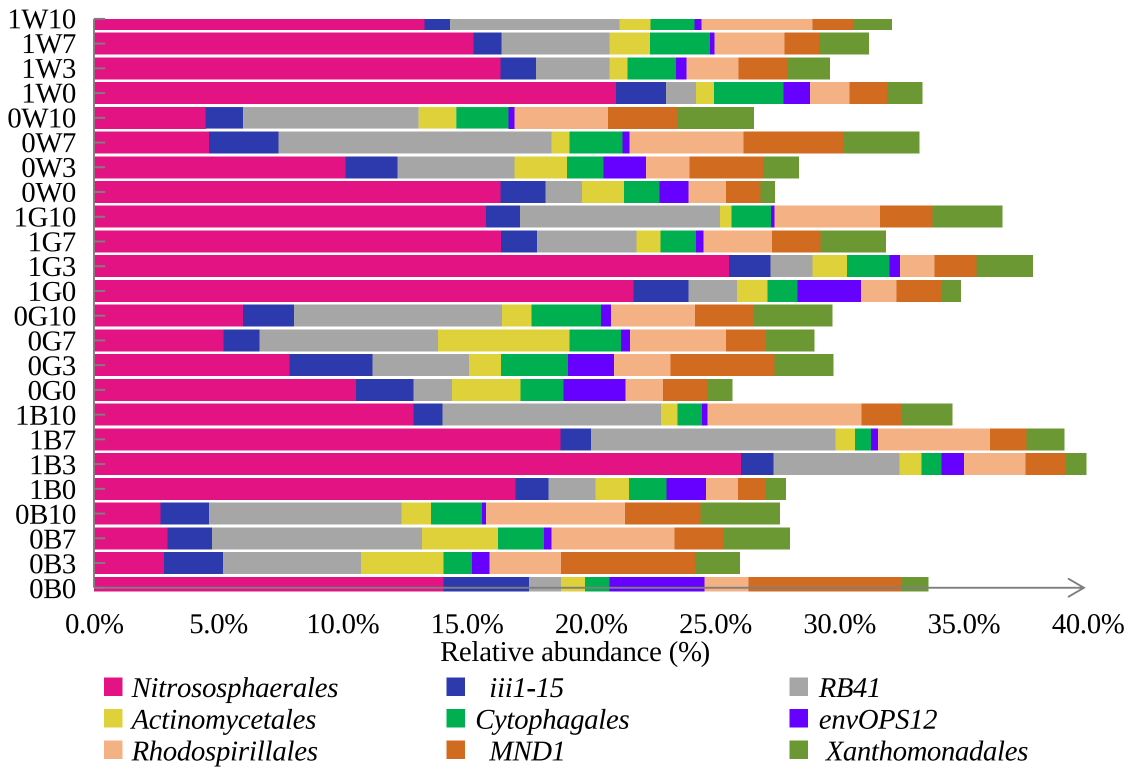

Fig. S2. Community structure in all samples at the orders level. (relative abundances (>0.2%) are based on the proportional frequencies of those DNA sequences that could be classified at the orders level.)

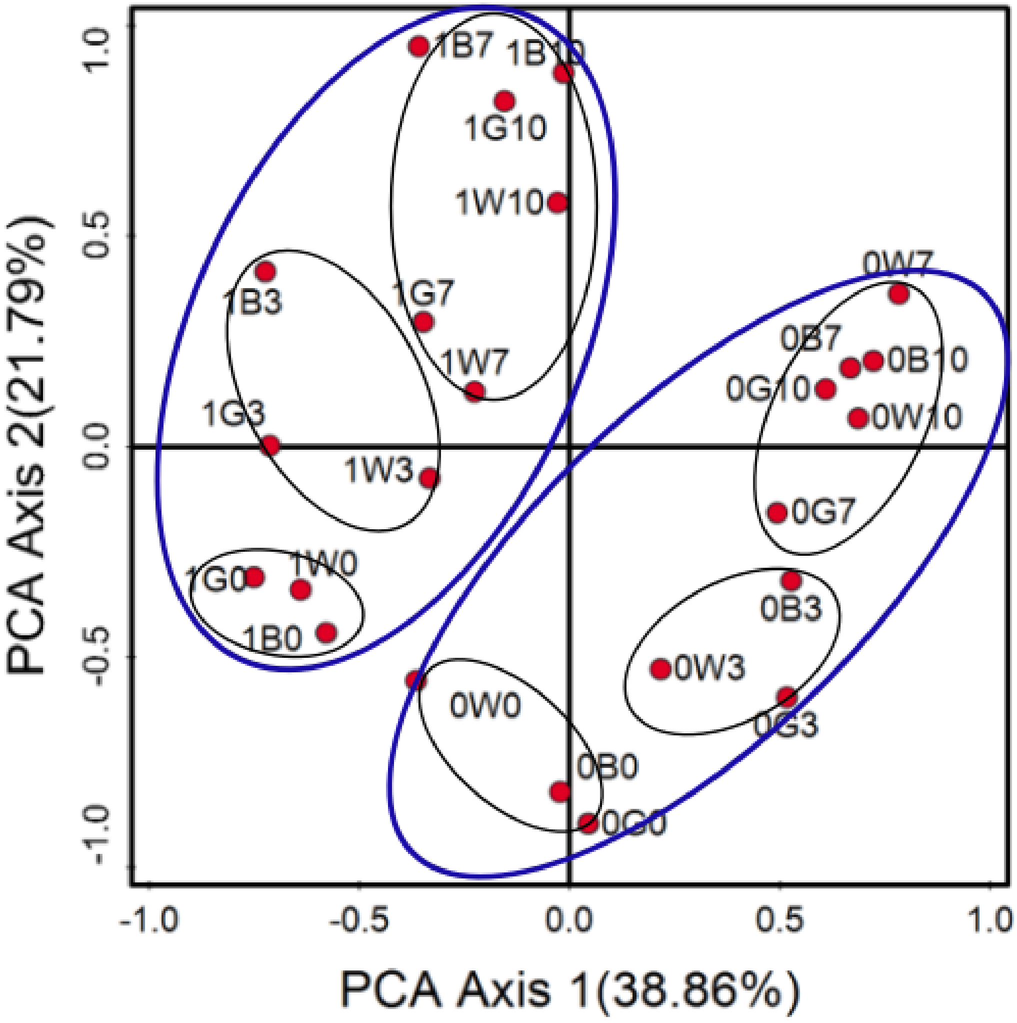

Fig. S3. Multiple-samples PCA analysis of 24 samples associated with bacterial community and physicochemical soil.

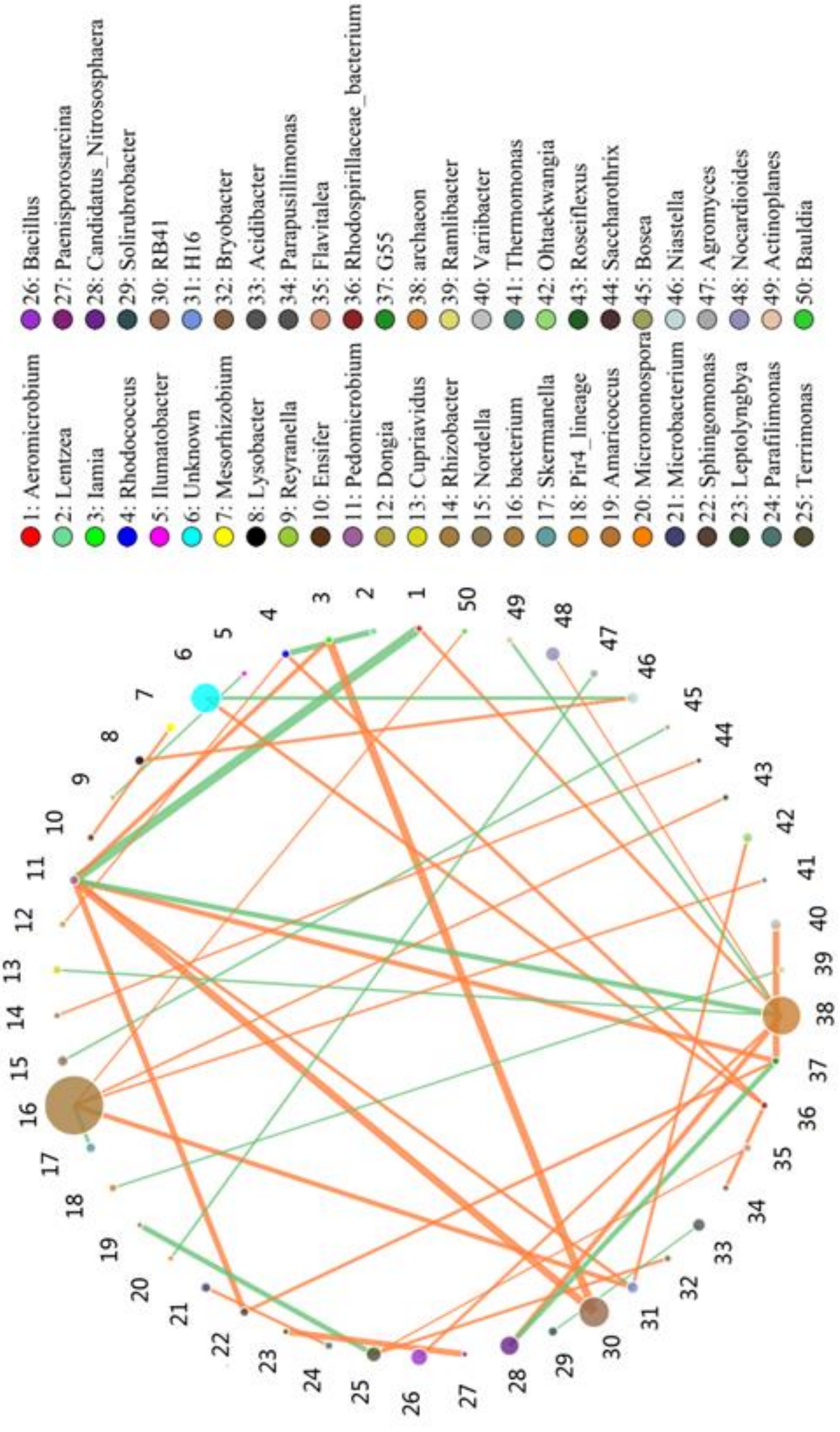

Fig. S4. Correlation network diagram at the genera level. (circles represent specifical species, and the size of circles stand for the abundance. the correlation between two species describe as line weight and color. The wide lines explained the strength of correlation. The color of lines is orange, which is positive correlation, and green stand for negative correlation. Screening data with a

correlation greater group (>0.1) draw a common expression analysis network diagram at the python level).
